# Robust Cooperative Multi-Vehicle Tracking with Inaccurate Self-Localization Based on On-Board Sensors and Inter-Vehicle Communication

**DOI:** 10.3390/s20113212

**Published:** 2020-06-05

**Authors:** Xiaobo Chen, Jianyu Ji, Yanjun Wang

**Affiliations:** 1 Automotive Engineering Research Institute, Jiangsu University, Zhenjiang 212013, China; 2 School of Automotive and Traffic Engineering, Jiangsu University, Zhenjiang 212013, China; 18852852561@163.com (J.J.); 18651403397@163.com (Y.W.)

**Keywords:** cooperative perception, multi-vehicle tracking, Bayes inference, track association

## Abstract

The fusion of on-board sensors and transmitted information via inter-vehicle communication has been proved to be an effective way to increase the perception accuracy and extend the perception range of connected intelligent vehicles. The current approaches rely heavily on the accurate self-localization of both host and cooperative vehicles. However, such information is not always available or accurate enough for effective cooperative sensing. In this paper, we propose a robust cooperative multi-vehicle tracking framework suitable for the situation where the self-localization information is inaccurate. Our framework consists of three stages. First, each vehicle perceives its surrounding environment based on the on-board sensors and exchanges the local tracks through inter-vehicle communication. Then, an algorithm based on Bayes inference is developed to match the tracks from host and cooperative vehicles and simultaneously optimize the relative pose. Finally, the tracks associated with the same target are fused by fast covariance intersection based on information theory. The simulation results based on both synthesized data and a high-quality physics-based platform show that our approach successfully implements cooperative tracking without the assistance of accurate self-localization.

## 1. Introduction

Nowadays, intelligent vehicles equipped with advanced driver assistance systems (ADASs) can perceive other road participants and obstacles, including vehicles, pedestrians, etc., through on-board sensors. A variety of on-board sensors have been widely applied to achieve this goal, such as camera, Lidar, Radar, and so on. The perception system [1,2] of intelligent vehicles captures the measurements of surrounding targets through these sensors and build an environmental model which reflects the real states of different targets.

Multi-vehicle tracking (or more generally, multi-object tracking, MOT) is a crucial perception task since an accurate estimate of surrounding vehicles plays an important role in subsequent collision avoidance and route planning. The main challenge in MOT is the determination of the association between measurements and targets. In the literatures, extensive algorithms have been proposed to handle the data association problem. Multiple hypothesis tracking (MHT) [3] is known as a powerful algorithm to address the data association problem in MOT. Although MHT is Bayesian optimal in theory, the exact solution of MHT is computationally intractable, thus, requiring proper approximation. Joint probabilistic data association (JPDA) [4] makes a soft association with the assumption that each measurement can originate from several candidate targets. JPDA can achieve reasonable results with lower computational burden. Recently, Mahler first used the Bayesian filter to derive a multi-target tracking algorithm based on random finite set (RFS) theory [5,6]. Under the RFS framework, the Bayes recursion formula for tracking single targets can be extended to multi-target tracking problems. The resulting probability hypothesis density (PHD) filter can propagate the PHD or the first-order moment of the multi-target posterior density. The integral of the PHD over a region in the state space gives the expected number of targets within this region, while the peaks in the PHD represents the state of the targets. To address the computationally intractable multiple integrals issue, Gaussian mixture PHD (GMPHD) [7] was further developed which led to a closed-form solution to the PHD filter recursion.

In reality, due to the physical mechanism, the on-board sensors of intelligent vehicles always suffer from inherent drawbacks, such as limited perception range or field-of-view (FOV). Moreover, in the crowd scenarios where frequent occlusion occurs, intelligent vehicles fail to detect the target that is occluded, thus, increasing the potential risk of traffic conflicts. To address the above issue, cooperative perception (or collaborative perception) [8,9,10] based on inter-vehicle communication has attracted much attention recently. For instance, dedicated short range communication (DSRC) has been introduced as a useful technique which allows vehicles to communicate with other neighboring vehicles through the on-board units (OBUs) installed in the vehicles. When the vehicles communicate successfully, they can exchange their respective local perception results. In fact, inter-vehicle communication (more strictly speaking, the wireless interface) can be viewed as a type of virtual sensor [11,12] in the sense that a host vehicle can combine its local estimate and a remote message transmitted from other cooperative vehicles to form a more accurate and complete environmental model. Due to the significant distance of inter-vehicle communication and the fusion of information from different viewpoints, cooperative perception not only increases the accuracy and perceptual range beyond line of sight, but also reduces blind spots caused by mutual occlusion, weather, and other external factors.

By introducing inter-vehicle communication into multi-object tracking, cooperative tracking has emerged as a promising technique. In [13], a collaborative sensor fusion algorithm was proposed for MOT by combing GMPHD filter and covariance intersection. The cooperative tracking algorithm was further applied to assist the overtaking system [14] and the results confirmed the advantage of cooperative sensing. In [15], a data association and fusion framework was proposed for multi-vehicle cooperative perception. An interactive multiple model (IMM) filter [16] was used to estimate the state of vehicles and Bhattacharyya distance was applied to measure the difference between local tracks from a host vehicle and communicated tracks from a cooperative vehicle. After association, fast covariance intersection (FCI) [17] which computed the weights directly without nonlinear optimization was employed to fuse together the tracks associated with the same target. This work relied on the assumption of on-board measurements available in global coordinate systems, which required the accurate self-localization of both host and cooperative vehicles. In [18], the temporal and spatial alignment between the local environment model of host and cooperative vehicles were reviewed, however, target association and track fusion were not discussed. Some works [19,20,21] studied the track-to-track association problem in the case of cooperative sensing under the assumption of accurate positioning information.

In summary, current cooperative multi-vehicle tracking methods typically assume the self-localization information, such as position and orientation, of both host and cooperative vehicles is accurate such that the local tracks perceived by two vehicles can be readily converted into either a global frame or the local frame of host vehicle by coordinate system transformation. Then, track association and fusion can be conducted and the information from cooperative vehicle can be used to enhance the perception performance of host vehicle. However, accurate self-localization through positioning systems, such as Global Positioning System (GPS), is not always accurate enough or even available [22]. For example, in dense urban environment where the vehicles are surrounded by skyscrapers or tall buildings, the location information provided by GPS would not be reliable. In such cases, inaccurate or even unavailable self-localization information seriously affects the performance of cooperative tracking system.

To address the above problem and enhance the robustness of cooperative multi-vehicle tracking, we propose, in this paper, an integrated framework which can determine the relative pose (including translation and orientation) of host and cooperative vehicles relying on their respective local tracks, instead of global positioning information. Moreover, this work concentrates on the dynamic situation where the relative translation and orientation between host and cooperative vehicles are changing with time. This is more consistent with real traffic environment where vehicles are usually driving with different intentions. Consequently, our cooperative multi-vehicle tracking system still works without the assistance of accurate self-localization information.

It should be emphasized that in the literature of wireless sensor networks (WSNs), some target tracking algorithms [23,24,25] have been developed for simultaneous localization and tracking (SLAT). In [24], a Bayesian filtering framework was proposed to infer the joint posterior distribution of both target and multiple sensors. Variational method [26] was used to approximate the joint state during the measurements correction stage. In [25], a dynamic non-parametric belief propagation (DNBP) method was proposed for cooperative vehicle sensing. However, most works in SLAT tend to track one target by using multiple static or moving sensors, thus, restricting their application to more complex scenarios where the number of targets can vary at different times.

The rest of this paper is organized as follows: In Section 2, we briefly review the adaptive GMPHD filter which is the basic component for target tracking; in Section 3, we present our framework of cooperative tracking and propose a Bayes model for simultaneous track association and relative pose estimation; in Section 4 we report some simulation results based on both the synthesized data and PreScan-based system; and finally, we provide some conclusions and future works in Section 5.

## 2. Adaptive GMPHD Filter for MOT

### 2.1. PHD Filter Formulation

In PHD filter, both the multi-target state and the set of measurements at each time step are represented by RFS. The PHD filter approximates the multi-target Bayes filter through propagating the first-order moment. The recursive process of PHD filter includes two steps, i.e., prediction and correction (or update) as follows:

1. PHD prediction
  (1) $$v_{k|k-1}\left(x\right)=\int \left[P_{S,k}\left(\zeta \right)f_{k|k-1}\left(x|\zeta \right)+\beta_{k|k-1}\left(x|\zeta \right)\right]v_{k-1|k-1}\left(\zeta \right)d\zeta +\gamma_{k}\left(x\right)$$
  where $v_{k|k-1}\left(x\right)$ and $v_{k-1|k-1}\left(\zeta \right)$ represent the predicted and posterior intensity of the target at time k−1, $P_{S,k}\left(\zeta \right)$ is the survival probability when the target state is ζ, $f_{k|k-1}\left(⬝|⬝\right)$ is the single-target state transition density, $\beta_{k|k-1}\left(⬝|⬝\right)$ and $\gamma_{k}\left(x\right)$ denote the intensity of spawning target and newborn target, respectively.
2. PHD correction
  (2) $$v_{k|k}\left(x\right)=\left[1-P_{D,k}\left(x\right)\right]v_{k|k-1}\left(x\right)+\underset{z\in Z^{\left(k\right)}}{\sum }\frac{P_{D,k}\left(x\right)g_{k}\left(z|x\right)v_{k|k-1}\left(x\right)}{\kappa_{k}\left(z\right)+\int P_{D,k}\left(\zeta \right)g_{k}\left(z|\zeta \right)v_{k|k-1}\left(\zeta \right)d\zeta }$$
  where $Z^{\left(k\right)}=\left\{z_{k,1},z_{k,2},\ldots ,z_{k,M_{k}}\right\}$ denotes the measurement set at time k, $M_{k}$ is the total number of measurements, $P_{D,k}\left(x\right)$ is the detection probability when the target state is x, $g_{k}\left(⬝|⬝\right)$ is the single-target measurement likelihood function, and $\kappa_{k}\left(⬝\right)$ is the intensity of the clutter RFS.

The above two formulas are the basic recursive equations for PHD filtering. After correction at each time, the expected number of targets can be obtained by integrating the updated PHD intensity, and then taking the nearest integer value. The status of each target can be obtained from the state corresponding to the updated PHD peak point. It can be seen that the PHD filter can avoid the data association problem. However, the PHD filter includes multiple integration operations, which cannot obtain an analytical solution, and suffer from “dimensional disaster” in the calculation of numerical integration.

### 2.2. Gaussian Mixture Implementation

In order to give a closed-form solution for PHD recursion, Gaussian mixture PHD (GMPHD) filter uses a set of Gaussian components to approximate the posterior density where the weights, mean values, and covariances of each Gaussian component are continuously updated over time. Suppose that the posterior intensity at time k−1 is expressed as follows:

(3) $$v_{k-1|k-1}\left(x\right)=\overset{J_{k-1|k-1}}{\underset{i=1}{\sum }}w_{k-1}^{\left(i\right)}N\left(x;m_{k-1}^{\left(i\right)},P_{k-1}^{\left(i\right)}\right)$$

where $J_{k-1|k-1}$ represents the number of Gaussian components at time k−1, N(x|a,B) stands for the multivariate Gaussian distribution with mean a and covariance B. It is assumed that transition probability density and observed likelihood function also follow Gaussian distribution as follows:

(4) $$f_{k|k-1}\left(x|\zeta \right)=N\left(x;F_{k-1}\zeta ,Q_{k-1}\right)$$

(5) $$g_{k}\left(z|x\right)=N\left(z;H_{k}x,R_{k}\right)$$

where $F_{k-1}$ and $H_{k}$ are linear state transition matrix and linear observation matrix, respectively, and $Q_{k-1}$ and $R_{k}$ are the covariance matrices of the process noise and the measurement noise, respectively.

Substituting $v_{k-1|k-1}\left(x\right)$ in Equation (3) into the PHD prediction and correction equations, we can obtain the recursive form of the PHD in the Gaussian mixture form. Specifically, GMPHD performs the prediction and correction as follows:

1. GMPHD prediction
  (6) $$v_{k|k-1}\left(x\right)=\overset{J_{k|k-1}}{\underset{i=1}{\sum }}w_{k|k-1}^{\left(i\right)}N\left(x;m_{k|k-1}^{\left(i\right)},P_{k|k-1}^{\left(i\right)}\right)$$
  In this work, the spawning target is ignored and the prediction Formula (6) can be rewritten as
  (7) $$v_{k|k-1}\left(x\right)=v_{S,k|k-1}\left(x\right)+\gamma_{k}\left(x\right)$$
  where
  (8) $$v_{S,k|k-1}\left(x\right)=P_{S,k}\overset{J_{k|k-1}}{\underset{i=1}{\sum }}w_{k-1}^{\left(i\right)}N\left(x;m_{S,k|k-1}^{\left(i\right)},P_{S,k|k-1}^{\left(i\right)}\right)$$
  (9) $$m_{S,k|k-1}^{\left(i\right)}=F_{k-1}m_{k-1}^{\left(i\right)}$$
  (10) $$P_{S,k|k-1}^{\left(i\right)}=F_{k-1}P_{k-1}^{\left(i\right)}F_{k-1}^{T}+Q_{k-1}$$
2. GMPHD correction
  (11) $$v_{k|k}\left(x\right)=\left(1-P_{D,k}\right)v_{k|k-1}\left(x\right)+\underset{z\in Z_{k}}{\sum }\overset{J_{k|k-1}}{\underset{j=1}{\sum }}w_{k}^{\left(j\right)}\left(z\right)N\left(x;m_{k|k}^{\left(j\right)},P_{k|k}^{\left(j\right)}\right)$$
  where
  (12) $$w_{k}^{\left(j\right)}\left(z\right)=\frac{P_{D,k}w_{k|k-1}^{\left(j\right)}N\left(x;H_{k}m_{k|k-1}^{\left(j\right)},H_{k}P_{k|k-1}^{\left(j\right)}H_{k}^{T}+R_{k}\right)}{\kappa_{k}\left(z\right)+P_{D,k}\sum_{l}^{J_{k|k-1}}w_{k|k-1}^{\left(l\right)}N\left(x;H_{k}m_{k|k-1}^{\left(l\right)},H_{k}P_{k|k-1}^{\left(l\right)}H_{k}^{T}+R_{k}\right)}$$
  (13) $$m_{k|k}^{\left(j\right)}=m_{k|k-1}^{\left(j\right)}+K_{k}^{\left(j\right)}\left(z-H_{k}m_{k|k-1}^{\left(j\right)}\right)$$
  (14) $$P_{k|k}^{\left(j\right)}=\left(I-K_{k}^{\left(j\right)}H_{k}P_{k|k-1}^{\left(j\right)}\right)$$
  (15) $$K_{k}^{\left(j\right)}=P_{k|k-1}^{\left(j\right)}H_{k}^{T}{\left(H_{k}P_{k|k-1}^{\left(j\right)}H_{k}^{T}+R_{k}\right)}^{-1}$$

After the GMPHD correction is completed, the Gaussian components with small weight are pruned and the Gaussian components close to each other are merged. Finally, in order to extract tracks, the mean value of the Gaussian component with the weight greater than a certain threshold is used as the multi-object state estimation.

For the application of GMPHD, the intensity of newborn target $\gamma_{k}\left(x\right)$ should be defined at each time, indicating the possible state when new targets appear. In our cases, the target vehicles can encounter the FOV of host and cooperative vehicles at different positions and times. As a result, it is infeasible to define $\gamma_{k}\left(x\right)$ in advance. To address the problem, at each time, we let $\gamma_{k}\left(x\right)$ be driven by the observed measurements adaptively as follows:

(16) $$\gamma_{k}\left(x\right)=\overset{M_{k}}{\underset{i=1}{\sum }}w^{\left(i\right)}N\left(x;{\bar{z}}_{k,i},P_{0}\right)$$

where $P_{0}$ denotes the initial covariance matrix and ${\bar{z}}_{k,i}$ denotes the state constructed from the measurement $z_{k,i}$ In such a way, the resulting adaptive GMPHD filter is applicable to the cooperative tracking situations we concern.

## 3. Cooperative Tracking with Inaccurate Self-Localization

### 3.1. Framework of Cooperative Tracking

**Figure 1:**
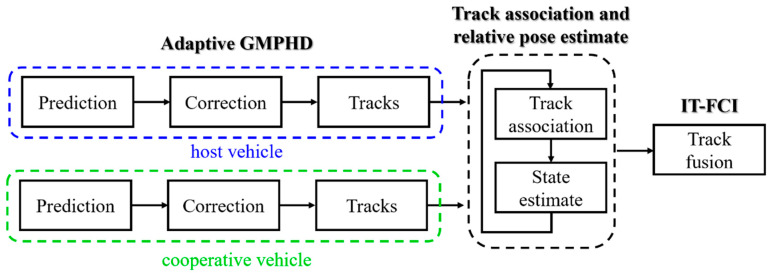
The proposed cooperative multi-vehicle tracking framework.

We assume that many vehicles are moving in the environment according to their maneuvers. Among these vehicles, some vehicles can independently sense the other vehicles within in certain range by using on-board sensors and exchange their local information via inter-vehicle communication. The vehicle transmitting message is called cooperative vehicle, whereas the vehicle receiving message and performing fusion is called host vehicle. Certainly, a vehicle can send or receive message depending on its role. The other vehicles not involved in cooperation are called target vehicles. This work concentrates on the situation where the relation translation and rotation between host and cooperative vehicles are dynamically changing and inaccurate (or even unknown), thus, preventing the direct application of existing technologies. To address the above problem and achieve sensor fusion for cooperative multi-vehicle tracking, we propose a novel framework depicted in Figure 1.

As shown in Figure 1, the host and cooperative vehicles first obtain respective local tracks by conducting the adaptive GMPHD algorithm presented in Section 2. Then, the cooperative vehicle transmits its local tracks to the host vehicle which attempts to estimate the relative translation and rotation between two vehicles and simultaneously associate the locals track from two vehicles by using the algorithm explained next. Finally, the matched tracks are fused following fast covariance intersection based on information theory (IT-FCI) [27].

### 3.2. Track Association and Relative Pose Estimation

#### 3.2.1. Formulation

**Figure 2:**
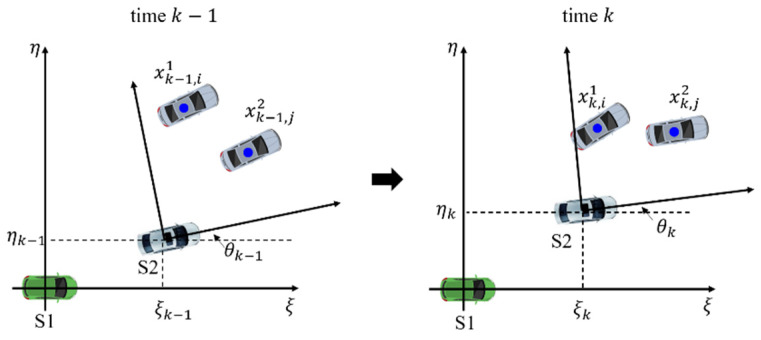
Relative translation and orientation between vehicle S1 and vehicle S2.

The relative pose estimation and track association problem in dynamic scenarios is shown in Figure 2. Here, we focus on two-dimensional space for brevity, however, our proposed method can be extended to higher-dimensional space with slight modification. Given two vehicles S1 and S2, here, S1 is assumed to be the host vehicle, and S2 is assumed to be the cooperative vehicle, indicating that S2 sends its local estimates to S1 where the information fusion is performed. At given time k, let $X_{k}^{1}=\left\{x_{k,1}^{1},x_{k,2}^{1},\cdots ,x_{k,N_{k}^{1}}^{1}\right\}$ and $X_{k}^{2}=\left\{x_{k,1}^{2},x_{k,2}^{2},\cdots ,x_{k,N_{k}^{2}}^{2}\right\}$ be the collection of local tracks of S1 and S2 which are obtained through MOT algorithm, such as adaptive GMPHD in Section 2. $N_{k}^{1}$ and $N_{k}^{2}$ denote the total number of tracks in S1 and S2, respectively. Moreover, the relative location and orientation of S2 with respect to S1 at time k is characterized by $s_{k}={\left[\xi_{k},{\dot{\xi }}_{k},\eta_{k},{\dot{\eta }}_{k},\theta_{k},{\dot{\theta }}_{k}\right]}^{T}$ where $\xi_{k}$ and $\eta_{k}$, denote the translation of S2 in the Cartesian coordinate system of S1. Similarly, $\theta_{k}$ denotes the orientation angle. ${\dot{\xi }}_{k}$, ${\dot{\eta }}_{k}$, and ${\dot{\theta }}_{k}$ represent the corresponding velocities.

Any track $x_{k,j}^{2}$ in the coordinate of S2 can be exactly transformed to that of S1 as follows:

(17) $$x_{k,j}^{2\to 1}=R\left(\theta_{k}\right)x_{k,j}^{2}+\left[\begin{array}{c} \xi_{k}\\ \eta_{k} \end{array}\right]$$

where $R\left(\theta_{k}\right)=\left[\begin{array}{cc} \cos\theta_{k} & -\sin\theta_{k}\\ \sin\theta_{k} & \cos\theta_{k} \end{array}\right]$ denotes the rotation matrix. In the situation we consider, a major difficulty is that $\left(\xi_{k},\eta_{k},\theta_{k}\right)$ is inaccurate (or even totally unknown) and dynamically changing with time. In addition, in the case of multiple targets, the association between tracks of different vehicles is also unknown.

Suppose the track association between two vehicles is denoted by the true but unknown $N_{k}^{1}\times N_{k}^{2}$ association matrix $U_{k}=\left\{u_{ij}^{k}\right\}$ with each entry $u_{ij}^{k}\in \left\{0,1\right\}$ representing the result of association between $x_{k,i}^{1}$ and $x_{k,j}^{2}$, $1\leq i\leq N_{k}^{1},1\leq j\leq N_{k}^{2}$. Formally, if $u_{ij}^{k}=1$, it means that local tracks $x_{k,i}^{1}$ and $x_{k,j}^{2}$ refer to the same target; otherwise, it means they belong to different target. Since it is assumed that each local track in one sensor corresponds to one, and at most one local track in other sensor, we have the constraints applied to $U_{k}$ below

(18) $$\sum_{j=1}^{N_{k}^{2}}u_{ij}^{k}\leq 1,\;\sum_{i=1}^{N_{k}^{1}}u_{ij}^{k}\leq 1,\;\forall 1\leq i\leq N_{k}^{1},1\leq j\leq N_{k}^{2}.$$

In addition, in order to incorporate the prior information about $s_{k}$, it is supposed that $s_{k}$ follows

(19) $$P\left(s_{k}\right)=N\left(s_{k}|a,B\right).$$

For example, similar to Kalman filter, we let $a=F{\hat{s}}_{k-1}$, $B=FP_{k-1}F^{T}+Q$, ${\hat{s}}_{k-1}$, and $P_{k-1}$ are the mean and covariance of $s_{k-1}$; F is the state transition matrix of cooperative vehicle; and Q is covariance matrix of the process noise.

Similarly, according to Equation (17), we have the following likelihood function:

(20) $$P\left(x_{k,i}^{1}|s_{k},x_{k,j}^{2}\right)=N\left(x_{k,i}^{1}|R\left(\theta_{k}\right)x_{k,j}^{2}+\left[\begin{array}{c} \xi_{k}\\ \eta_{k} \end{array}\right],\Sigma \right)$$

where Σ is the measurement noise covariance. The above assumption is reasonable, since $x_{k,j}^{2\to 1}$ should be close to $x_{k,i}^{1}$ when $x_{k,i}^{1}$ and $x_{k,j}^{2}$ refer to the same target. We also assume that the local tracks are independent of each other. On the basis of the above discussion, we propose the following probabilistic model:

(21) $$P\left(s_{k},X_{k}^{1},X_{k}^{2}|U_{k}\right)=P\left(s_{k}\right)\underset{j}{\prod }P\left(x_{k,j}^{2}\right)\underset{i,j}{\prod }P{\left(x_{k,i}^{1}|s_{k},x_{k,j}^{2}\right)}^{u_{ij}^{k}}$$

Other prior knowledge, for example, given noisy measurement of partial entries of $s_{k}$, can be easily integrated into Equation (21) by introducing extra likelihood function.

#### 3.2.2. Expectation-Maximum (EM) Solution Algorithm

We treat $s_{k},X_{k}^{1},X_{k}^{2},\;\mathrm{and}\;U_{k}$ as complete data; $X_{k}^{1},X_{k}^{2}$ as incomplete data; $s_{k}$ as hidden variable; and $U_{k}$ as unknown parameter. Then, we develop an effective solution in the spirit of the expectation-maximum (EM) solution algorithm, which attempts to jointly estimate the distribution of hidden variable $s_{k}$ and track association matrix $U_{k}$ in an iterative fashion. Specifically, the algorithm consists of the following two steps:

1. E-step
  (22) $$Q\left(U,U^{p-1}\right)=E\left\{logP\left(s_{k},X_{k}^{1},X_{k}^{2}|U\right)|U^{p-1}\right\};$$
2. M-step
  (23) $$U^{p}=\mathrm{argmax}Q\left(U,U^{p-1}\right).$$
  where p refers to the pth iteration of the algorithm. The E-step and M-step repeat until certain convergence criteria is satisfied.

##### E-Step

Given the estimation of $U_{k}$ at the (p−1)-th iteration, we need to calculate the expectation of $logP\left(s_{k},X_{k}^{1},X_{k}^{2}|U\right)$. Let $\Omega_{k}^{p-1}=\left\{\left(i,j\right)|u_{ij}^{k,p-1}=1\right\}$, indicating those associated tracks between sensors. Then, according to Bayes theorem and Equation (21), we can have the posterior distribution of $s_{k}$ as follows:

(24) $$P\left(s_{k}|X_{k}^{1},X_{k}^{2},U^{p-1}\right)=\frac{P\left(s_{k}\right)\prod_{\left(i,j\right)\in \Omega_{k}^{p-1}}P\left(x_{k,i}^{1}|s_{k},x_{k,j}^{2}\right)}{\int P\left(s_{k}\right)\prod_{\left(i,j\right)\in \Omega_{k}^{p-1}}P\left(x_{k,i}^{1}|s_{k},x_{k,j}^{2}\right)ds_{k}}$$

Considering that $R\left(\theta_{k}\right)$ in the above distribution is nonlinear with respect to $s_{k}$, we apply Talyor series expansion to obtain the first-order linear approximation around current estimate $\theta_{k}^{l-1}$ as follows:

(25) $$R\left(\theta_{k}\right)x_{k,j}^{2}+\left[\begin{array}{c} \xi_{k}\\ \eta_{k} \end{array}\right]\approx H\left(\theta_{k}^{l-1}\right)s_{k}+R\left(\theta_{k}^{l-1}\right)x_{k,j}^{2}-\theta_{k}^{l-1}\bar{R}\left(\theta_{k}^{l-1}\right)x_{k,j}^{2}$$

where $\bar{R}\left(\theta_{k}\right)=\left[\begin{array}{cc} -\sin\theta_{k} & -\cos\theta_{k}\\ \cos\theta_{k} & -\sin\theta_{k} \end{array}\right]$ is the Jacobian matrix of $R\left(\theta_{k}\right)$ evaluated at $\theta_{k}$, $H\left(\theta_{k}^{l-1}\right)$ is defined as

(26) $$H\left(\theta_{k}^{l-1}\right)=\left[\begin{array}{cc} \begin{array}{ccc} 1 & 0 & 0 \end{array} & \begin{array}{ccc} 0 & -x_{k,j}^{2}\left(1\right)\sin\theta_{k}^{l-1}-x_{k,j}^{2}\left(2\right)\cos\theta_{k}^{l-1} & 0 \end{array}\\ \begin{array}{ccc} 0 & 0 & 1 \end{array} & \begin{array}{ccc} 0 & x_{k,j}^{2}\left(1\right)\cos\theta_{k}^{l-1}-x_{k,j}^{2}\left(2\right)\sin\theta_{k}^{l-1} & 0 \end{array} \end{array}\right]$$

where $x_{k,j}^{2}={\left[x_{k,j}^{2}\left(1\right),x_{k,j}^{2}\left(2\right)\right]}^{T}$.

Incorporating Equation (25) into Equation (20), the likelihood can be approximated as

(27) $$P\left(x_{k,i}^{1}|s_{k},x_{k,j}^{2}\right)\approx N\left(x_{k,i}^{1}|H\left(\theta_{k}^{l-1}\right)s_{k}+R\left(\theta_{k}^{l-1}\right)x_{k,j}^{2}-\theta_{k}^{l-1}\bar{R}\left(\theta_{k}^{l-1}\right)x_{k,j}^{2},\Sigma \right).$$

Noticing the cumulative product in the numerator of Equation (24), we can, thus, iteratively apply each likelihood function $P\left(x_{k,i}^{1}|s_{k},x_{k,j}^{2}\right)$ to update the distribution of $s_{k}$. For instance, given any $\left(i,j\right)\in \Omega_{k}^{p-1}$, we have

(28) $$N\left(s_{k}|a,B\right)N\left(x_{k,i}^{1}|R\left(\theta_{k}\right)x_{k,j}^{2}+\left[\begin{array}{c} \xi_{k}\\ \eta_{k} \end{array}\right],\Sigma \right)\propto cN\left(s_{k}|a^{\prime },B^{\prime }\right)$$

where

(29) $$a^{\prime }=a+K\left(x_{k,i}^{1}-R\left(\theta_{k}^{l-1}\right)x_{k,j}^{2}+\theta_{i}\bar{R}\left(\theta_{k}^{l-1}\right)x_{k,j}^{2}-H\left(\theta_{k}^{l-1}\right)a\right)$$

(30) $$B^{\prime }=\left(I-KH\left(\theta_{k}^{l-1}\right)\right)B$$

where I is a identity matrix and K is the Kalman gain defined by

(31) $$K=BH{\left(\theta_{k}^{l-1}\right)}^{T}{\left(H\left(\theta_{k}^{l-1}\right)BH{\left(\theta_{k}^{l-1}\right)}^{T}+\Sigma \right)}^{-1}.$$

Since c is a constant irrelevant to $s_{k}$, the above correction equations mean that the posterior distribution of $s_{k}$ after combing with likelihood $P\left(x_{k,i}^{1}|s_{k},x_{k,j}^{2}\right)$, also follows Gaussian distribution with updated mean $a^{\prime }$ and covariance $B^{\prime }$. As a result, by replacing the original a and B in Equation (19) with the latest estimates $a^{\prime }$ and $B^{\prime }$, the above correction procedure can repeat until all $\left(i,j\right)\in \Omega_{k}^{p-1}$ have been used to generate the final posterior distribution of $s_{k}$.

Finally, the conditional expectation of $logP\left(s_{k},X_{k}^{1},X_{k}^{2}|U\right)$ can be calculated as

(32) $$Q\left(U,U^{p-1}\right)\propto \sum_{i,j}u_{ij}E\left\{logP\left(x_{k,i}^{1}|s_{k},x_{k,j}^{2}\right)|U^{p-1}\right\}.$$

From Equation (20), we can see that the conditional expectation in Equation (32) is difficult to evaluate because $P\left(x_{k,i}^{1}|s_{k},x_{k,j}^{2}\right)$ is nonlinear with respect to $\theta_{k}$. Considering that $s_{k}$ follows Gaussian distribution, a special case of Monte Carlo (MC) approximation method [28] which uses the mean of $s_{k}$ is applied. Therefore, we obtain

(33) $$Q\left(U,U^{p-1}\right)\propto \underset{i,j}{\sum }u_{ij}r_{ij}^{T}\Sigma^{-1}r_{ij}$$

where $r_{ij}=x_{k,i}^{1}-\left[\begin{array}{cc} \cos{\hat{\theta }}_{k} & -\sin{\hat{\theta }}_{k}\\ \sin{\hat{\theta }}_{k} & \cos{\hat{\theta }}_{k} \end{array}\right]x_{k,j}^{2}-\left[\begin{array}{c} {\hat{\xi }}_{k}\\ {\hat{\eta }}_{k} \end{array}\right]$, ${\hat{\theta }}_{k}$,$\;{\hat{\xi }}_{k}$, and ${\hat{\eta }}_{k}$ denote the estimated entries of $s_{k}$.

##### M-Step

In the M-step, the association matrix U needs to be updated by solving the following constrained optimization problem:

(34) $$\begin{array}{c} U^{p}=\mathrm{argmax}Q\left(U,U^{p-1}\right)\propto \underset{i,j}{\sum }u_{ij}d_{ij}^{2}\\ s.t.\;u_{ij}\in \left\{0,1\right\},\sum_{j=1}^{N_{k}^{2}}u_{ij}\leq 1,\;\sum_{i=1}^{N_{k}^{1}}u_{ij}\leq 1 \end{array}$$

where $d_{ij}^{2}=r_{ij}^{T}\Sigma^{-1}r_{ij}$ is the Mahalanobis distance between local tracks $x_{k,i}^{1}$ and $x_{k,j}^{2\to 1}$. We notice that Equation (34) is a linear sum assignment problem (LSAP) which can be readily solved by the Hungarian algorithm in polynomial time [29]. In addition, for local tracks corresponding to the same target, $d_{ij}^{2}$ should be small, otherwise $d_{ij}^{2}$ should be large. Taking these into account, an extra thresholding step is applied to $d_{ij}^{2}$ such that local tracks with large distance are removed from association.

### 3.3. Track Fusion

The last stage of our proposed framework is to combine different estimates (generated by host and cooperative vehicles, respectively) of the same target vehicle into one solution. Since it is difficult to calculate the cross-correlation among multiple estimates, especially for our distributed fusion architecture, direct application of optimal Bayes fusion can lead to overconfidence [30]. To address this problem, we apply a special version of covariance intersection (CI), termed as information theory based fast CI (IT-FCI) [27] which is given as:

(35) $${\hat{x}}_{FCI}=P_{FCI}\left(\omega P_{1}^{-1}{\hat{x}}_{1}+\left(1-\omega \right)P_{2}^{-1}{\hat{x}}_{2}\right)$$

(36) $$P_{FCI}^{-1}=\omega P_{1}^{-1}+\left(1-\omega \right)P_{2}^{-1}$$

where $\left({\hat{x}}_{1},P_{1}\right)$ and $\left({\hat{x}}_{2},P_{2}\right)$ denote two estimates of state corresponding to the same target, ω is the weight. Let l denote the dimension of state, then ω is determined by

(37) $$\omega =\frac{D\left(p_{1},p_{2}\right)}{D\left(p_{1},p_{2}\right)+D\left(p_{2},p_{1}\right)}$$

(38) $$D\left(p_{i},p_{j}\right)=\frac{1}{2}\left[\ln\frac{\left|P_{j}\right|}{\left|P_{i}\right|}+\sqrt{{\left({\hat{x}}_{i}-{\hat{x}}_{j}\right)}^{T}P_{j}^{-1}\left({\hat{x}}_{i}-{\hat{x}}_{j}\right)}+tr\left(P_{i}P_{j}^{-1}\right)-l\right].$$

## 4. Performance Evaluation and Results

Currently, it is not easy to test cooperative perceptions system using real vehicles due to the high cost and potential risk [31]. Therefore, following previous studies [12], in this section, we carry out two types of computer simulation to evaluate the performance of the proposed cooperative multi-vehicle tracking.

### 4.1. Simulation Based on Synthesized Data

**Figure 3:**
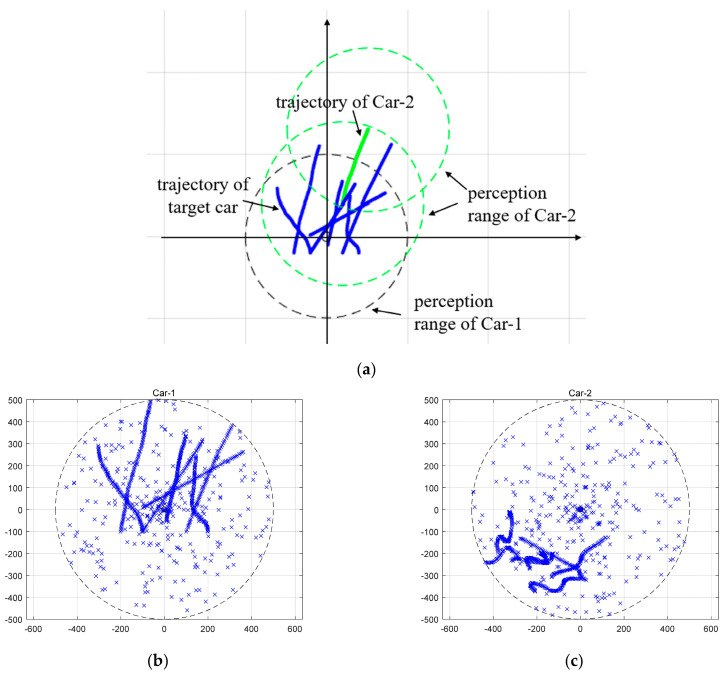
(**a**) Trajectories of target vehicles and Car-2 in the coordinate system of Car-1; (**b**) Measurements perceived by Car-1; and (**c**) Measurements perceived by Car-2.

A total of seven target vehicles are moving on the region (−800 m, 800 m) × (−800 m, 800 m). In addition, there are two intelligent vehicles (Car-1 and Car-2) which are equipped with sensor, and thus can sense the target vehicles in the environment. Car-1 and Car-2 is treated as the host and cooperative vehicle, respectively. Therefore, the local tracks from Car-2 are sent and fused with local tracks from Car-1 to enhance the perception performance. The perception range for each sensor is 500 m, indicating that the target vehicles with distance larger than 500 from Car-1 and Car-2 cannot be tracked. The coordinate system of Car-1 is viewed as reference system and the relative motion between the target vehicles and Car-1 is assumed to follow the near constant velocity (NCV) [32] motion model

(39) $$x_{k}=diag\left[F,F\right]x_{k-1}+diag\left[G,G\right]v_{k}$$

where diag represents a diagonal matrix, target state $x_{k}={\left[\xi_{k},{\dot{\xi }}_{k},\eta_{k},{\dot{\eta }}_{k}\right]}^{T}$, $F=\left[\begin{array}{cc} 1 & T\\ 0 & 1 \end{array}\right]$, $G=\left[\begin{array}{cc} T^{4}/4 & T^{3}/2\\ T^{3}/2 & T^{2} \end{array}\right]$, and $v_{k}\sim N\left(0,\sigma^{2}\right)$ with σ=0.5. For each target vehicle, the position $\xi_{k}$ and $\eta_{k}$ can be observed with measurement noise following Gaussian distribution with zero mean and covariance matrix diag[1,1]. For Car-2, besides the above relative motion in position, the relative rotation angle (in radian) between Car-2 and Car-1 also changes according to nonlinear model $\theta_{k}=0.3+0.1\sin\left(0.1k\right)$ in order to simulate the dynamic variation of vehicle heading. False alarms at any scan time are generated by Poisson distribution with mean λ=3. The probability of detection $P_{D}=0.98$. Adaptive GMPHD filtering algorithm, presented in Section 2, is conducted on Car-1 and Car-2 independently so that the local tracks can be obtained. The simulation was performed for 50 Monte Carlo runs with randomly generated process noise and measurements, thus, changing the trajectory and measurement of each target at each run. The simulation length is set to 100 s. Figure 3 show one simulation run. Figure 3a shows the relative trajectories of seven target vehicles and Car-2 in the coordinate system of Car-1. Figure 3b,c show the measurements from both the target vehicles and false alarms in the coordinate system of Car-1 and Car-2, respectively. As can be seen from Figure 3, at different times, Car-1 and Car-2 can track a different number of target vehicles because of vehicle appearance, disappearance, or out of perception range. The uncertain miss detection, i.e., false alarm, will influence the measurements observed by Car-1 and Car-2.

**Figure 4:**
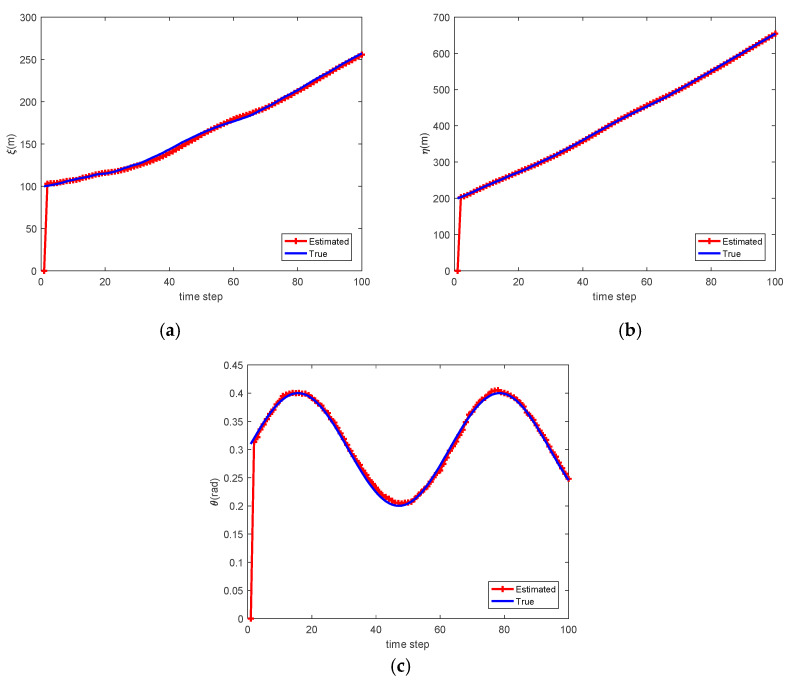
Estimate of (**a**) relative translation in *x*-axis; (**b**) relative translation in *y*-axis; (**c**) relative orientation.

**Figure 5:**
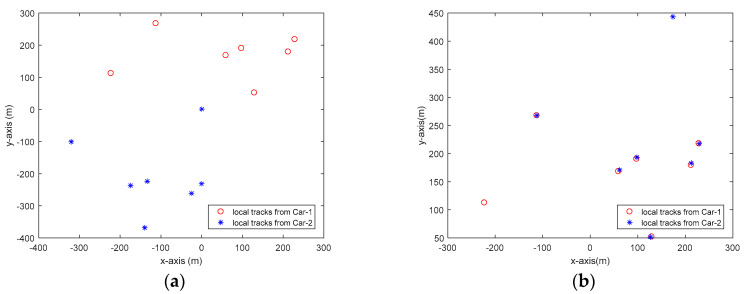
Illustration of local tracks from Car-1 and Car-2 at time step k=57. (**a**) Before association; (**b**) After association.

Figure 4 shows the estimate of relative translation and orientation angle in a simulation run. As can be seen, the estimated results are rather close to the true state of Car-2 with respect to Car-1. The association between local tracks from Car-1 and Car-2 at time k=57 is shown in Figure 5. To quantitatively measure the accuracy of state estimation of cooperative vehicle, we calculate the absolute error (AE) for the state estimation. For example, the AE for ξ is defined as follows:

(40) $$E_{\xi }=\frac{1}{K}\underset{k}{\sum }\left|\xi_{k}-{\hat{\xi }}_{k}\right|$$

where K=100 is the simulation length, $\xi_{k}$ and ${\hat{\xi }}_{k}$ represent the estimated and true position of Car-2 at time k. Finally, we calculate the average, maximum, and minimum AE across all Monte Carlo runs. The overall results are shown in Table 1 showing that state estimation is rather accurate.

To evaluate the effect of cooperative tracking, we use the criterion known as optimal subpattern assignment (OSPA) distance because it captures the difference in cardinality and individual elements between to finite sets [33,34]. The OSPA distance with order p and cut-off c is given by:

(41) $$d_{p}^{c}\left[X_{k},{\hat{X}}_{k}\right]={\left(\frac{1}{{\hat{N}}_{k}}\left(\underset{\pi \left(n\right)\in \Pi }{\min}\overset{N_{k}}{\underset{n=1}{\sum }}\min{\left(c,\|x_{k}^{n}-{\hat{x}}_{k}^{\pi \left(n\right)}{\|}_{2}\right)}^{p}+c^{p}\left|{\hat{N}}_{k}-N_{k}\right|\right)\right)}^{1/p}$$

where $X_{k}=\left\{x_{k}^{1},x_{k}^{2},\cdots ,x_{k}^{N_{k}}\right\}$,${\hat{X}}_{k}=\left\{{\hat{x}}_{k}^{1},{\hat{x}}_{k}^{2},\cdots ,{\hat{x}}_{k}^{{\hat{N}}_{k}}\right\}$, Π denotes all possible permutation of set $\left\{1,2,\cdots ,{\hat{N}}_{k}\right\}$. The above definition is suitable when $N_{k}\leq {\hat{N}}_{k}$, otherwise we should use $d_{p}^{c}\left[{\hat{X}}_{k},X_{k}\right]$.

**Figure 6:**
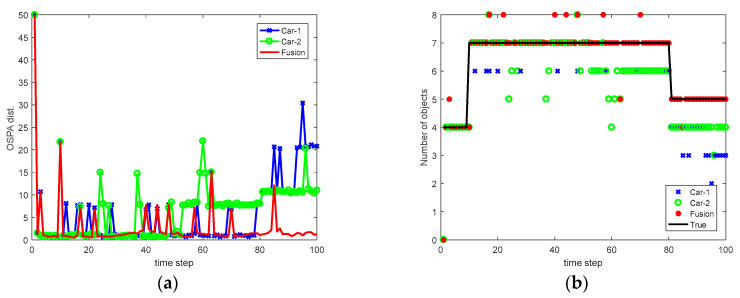
Results in a simulation run (**a**) variation of OSPA distance and (**b**) variation of the number of objects.

In the simulation, we let p=1 and c=50. The Monte Carlo average of the OSPA distance obtained by Car-1, Car-2, and the fusion are shown in Table 2. In addition, we also show in Table 2 the optimal fusion result (fusion-opt), assuming the relative translation and orientation is accurately given. As can be seen, compared with independent perception, the average OSPA obtained by sensor fusion (cooperative tracking) has reduced by 27% and 43% with respect to Car-1 and Car-2, respectively. The variation of OSPA distance and the number of objects versus time in a simulation run is shown in Figure 6. As we can observe, the local tracks from Car-1 and Car-2 can be fused, thus, reducing the OSPA distance and improving the performance of MOT.

In order to compare the CPU time when dealing with an oncoming set of measurements, we show in Table 3 the average execution time (millisecond) required by Car-1, Car-2, and the fusion stage. Notice that the execution time of Car-1 and Car-2 is mainly consumed by adaptive GMPHD filtering, while fusion stage concentrates on local tracks association and covariance intersection fusion. We can see from Table 3 that the execution time consumed by sensor fusion is less than 10% of the adaptive GMPHD filtering. It indicates that the introduction of sensor fusion does not influence the efficiency, however, significantly improves the tracking performance of the whole tracking system.

### 4.2. Simulation Based on PreScan Platform

**Figure 7:**
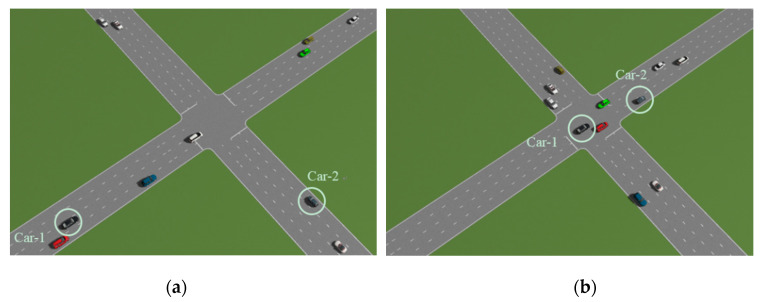
PreScan simulation. (**a**) Starting time; (**b**) Ending time.

**Figure 8:**
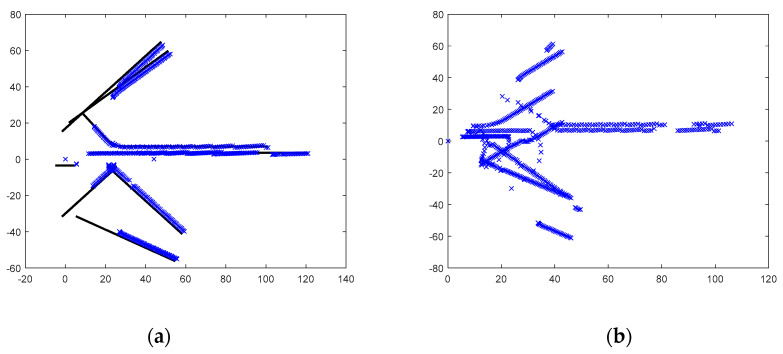
Illustration of observations. (**a**) Measurements and trajectory observed by Car-1; (**b**) Measurements observed by Car-2.

PreScan is a physics-based simulation platform which can be used to construct various driving environments for the design and verification of autonomous vehicles. In PreScan, a lot of elements, such as road, vehicles, sensors, vehicle-to-vehicle communication, weather etc., can be configured according to specific requirements. In order to evaluate our proposed cooperative multi-vehicle tracking system, we have built a driving scenario where 11 vehicles are deployed. Among these vehicles, two vehicles (called Car-1 and Car-2) are equipped with Radar sensor to percept surrounding vehicles. Table 4 shows the parameter of Radar. We can see from this table that for Car-1 and Car-2, only the leading vehicles with distance less than 150 m and azimuth in the range of −120° and 120° can be detected. The simulation length is 100. The simulation scenario at starting time and ending time is shown in Figure 7a,b, respectively. Car-1 and Car-2 are also marked in Figure 7 for clarity. Similar to the previous simulation, Car-1 is treated as the host vehicle, while Car-2 is treated as the cooperative vehicle. From Figure 7, we notice that both the relative translation and rotation between Car-1 and Car-2 are changing with time, especially when the two vehicles pass through the junction. In Figure 8, we illustrate the measurements observed by Car-1 and Car-2 in their respective coordinate system. For Car-1, we also show the true relative trajectories of the other vehicles. It can be observed that due to the limitation of perception range and possible occlusion between vehicles, some vehicles cannot always be detected during the simulation. Consequently, the measurements of certain vehicle cannot cover the corresponding trajectory completely.

**Figure 9:**
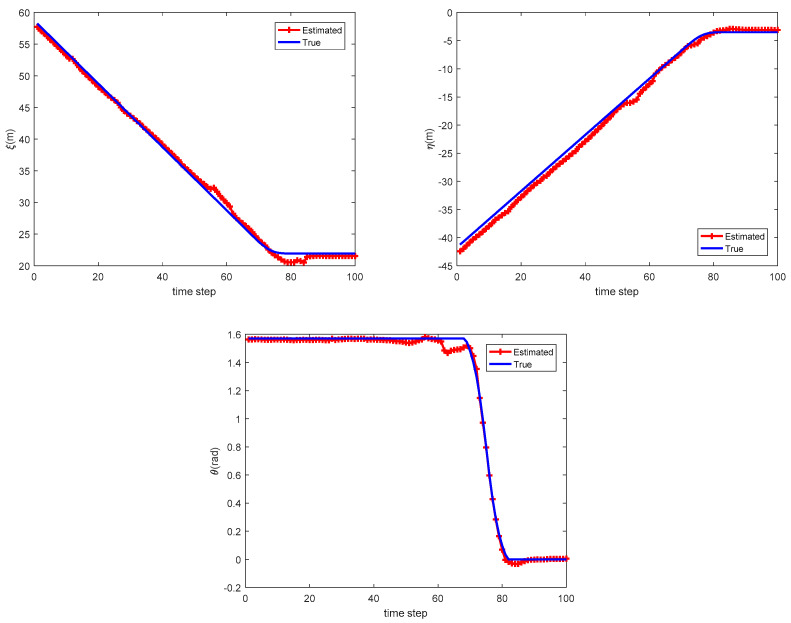
Estimate of relative translation and orientation.

**Figure 10:**
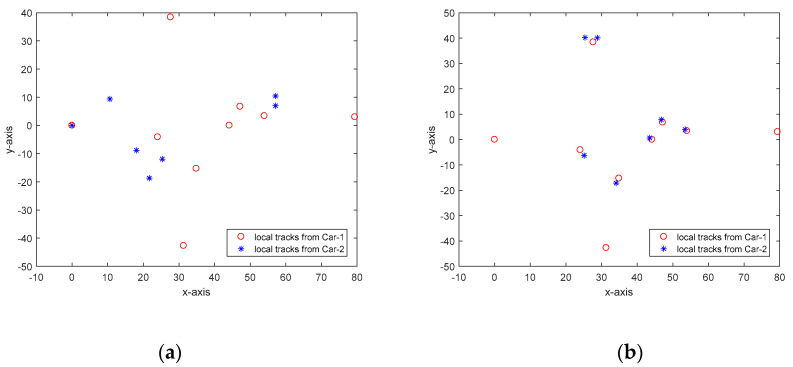
Illustration of local tracks from Car-1 and Car-2 at time step k=50. (**a**) Before association; (**b**) After association.

**Figure 11:**
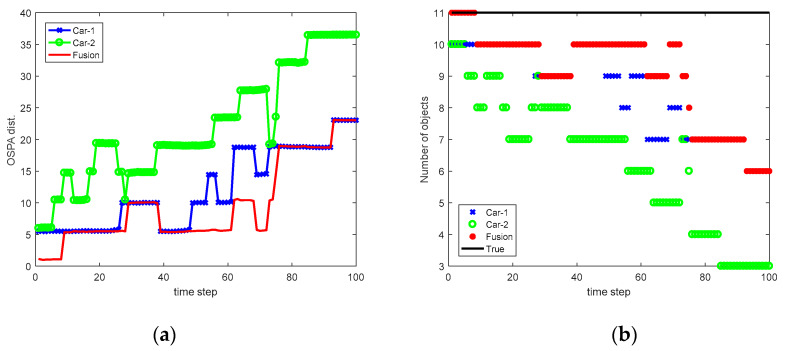
Results in a simulation run. (**a**) Variation of OSPA distance; (**b**) Variation of the number of objects.

The relative translation and orientation estimated based on our proposed approach are shown in Figure 9. We can see that in most cases, the estimated relative translation and orientation is rather close to the true value. Figure 10 shows the local tracks from two vehicles before and after association at time step k=50. In this case, Car-1 and Car-2 can detect nine and seven vehicles, respectively. After association, local tracks from Car-1 and Car-2 can be correctly matched, thus, leading to a total of 10 vehicles being detected. The variation of OSPA distance and the number of targets versus the simulation time is shown in Figure 11. The mean OSPA distance for Car-1, Car-2, and Fusion is 12.13, 22.10, and 9.80, respectively. A comparison with the case where Car-1 and Car-2 perform tracking alone shows that the fusion of their perception results not only reduces OSPA distance but also leads to better estimation of the number of vehicles. In summary, we conclude that the cooperative tracking successfully extends the perception field of view, thus, achieving superior MOT performance.

## 5. Concluding Remarks and Future Work

In this paper, we present a novel framework for cooperative multi-vehicle tracking when the self-localization information is not available. The adaptive GMPHD filter is applied to implement effective vehicle tracking when the intensity of newborn target is infeasible to define in advance. A Bayes formulation for joint track association and relative pose estimation is developed and the solution is derived by following the EM algorithm. Finally, the tracks associated successfully are fused by fast covariance intersection based on theory information. The simulation results demonstrate the relative pose between host and cooperative vehicles can be inferred accurately in most cases. In addition, with slightly increased computational costs, the cooperative multi-vehicle tracking demonstrates clear advantage over the non-cooperative tracking algorithm in terms of perception performance.

In reality, communication delay is another important factor that affects the performance of cooperative perception [35], especially when the bandwidth of the wireless channel is limited. Therefore, suitable temporal alignment is necessary to account for the time bias caused by the communication delay and algorithm execution. The simplest approach is based on the prediction model for the compensation of communication delay. This work assumes the tracks from different vehicles have been synchronized to the same time instant before track association and relative pose estimation. In addition, loss of communication links also hinders the application of our proposed algorithm. Actually, when the communication links interrupt, the host vehicle cannot receive the message from the cooperative vehicle, and thus fail to enhance the perception ability by fusion. After the communication links recover, the proposed algorithm can be performed by the host vehicle once the message from cooperative vehicle arrives. In future work, we plan to investigate the integration of temporal alignment into our framework to further enhance the performance of multi-vehicle tracking. Moreover, the extension of our approach to explicitly consider the effect of communication delays and failure is an interesting direction. One possible solution is to integrate these factors into our probabilistic model by introducing new variables. We would discuss these problems in future work.

## Figures and Tables

**Table 1:** Absolute error (AE) of state estimation.

State	Average AE	Maximum AE	Minimum AE
ξ	2.8330	3.4499	2.2454
η	3.4710	4.2924	2.9480
θ	0.0071	0.0090	0.0059

**Table 2:** Optimal subpattern assignment (OSPA) distance for multi-object tracking (MOT).

Method	Average OSPA	Maximum OSPA	Minimum OSPA
Car-1	3.992	5.926	2.641
Car-2	5.086	7.130	2.763
Fusion	2.896	3.937	1.930
Fusion-opt	2.063	2.662	1.583

**Table 3:** Execution time (ms) for MOT.

Method	Average Time	Maximum Time	Minimum Time
Car-1	32.86	35.01	31.43
Car-2	34.69	42.37	30.91
Fusion	2.64	3.19	2.39

**Table 4:** Radar sensor parameter configuration.

Parameter	Value
Scan pattern	Left to Right/Top to Bottom
Sweep rate	20 Hz
Beam range	150 m
Beam	120 deg
Beam	120 deg
